# Systems modelling as an approach for understanding and building consensus on non-communicable diseases (NCD) management in Cambodia

**DOI:** 10.1186/s12913-018-3830-2

**Published:** 2019-01-03

**Authors:** John P. Ansah, Amina Mahmood Islam, Victoria Koh, Vanthy Ly, Hero Kol, David B. Matchar, Chhun Loun, Mondol Loun

**Affiliations:** 1 Health Services and Systems Research, Duke-NUS Medical School Singapore, 8 College Road, Singapore, 169857 Singapore; 2 Centers for Disease Control and Prevention (CDC), Phnom Penh, Cambodia; 3 Ministry of Health Cambodia, Phnom Penh, Cambodia; 4 Department of Medicine, Duke University School of Medicine, Durham, NC USA

**Keywords:** Non-communicable diseases, Group model building, Simulation, Cambodia

## Abstract

**Background:**

This paper aims to demonstrate how systems modeling methodology of Group Model Building (GMD) can be applied for exploring and reaching consensus on non-communicable disease (NCD) management. This exercise was undertaken as a first step for developing a quantitative simulation model for generating credible estimates to make an investment case for the prevention and management of NCDs.

**Methods:**

Stakeholder engagement was facilitated through the use of a Group Model Building (GMB) approach. This approach combines various techniques in order to gain a whole system perspective.

**Results:**

A conceptual qualitative model framework that connects prevention—via risk factors reduction—screening and treatment of non-communicable diseases (NCDs) was developed with stakeholders that draws on stakeholders personal experiences, beliefs, and perceptions through a moderated interactions to gain in-depth understanding of NCDs management.

**Conclusion:**

Managing NCDs in Cambodia will require concerted effort to tackle NCD risk factors, identifying individuals with NCDs through screening and providing adequate and affordable consistent care to improve health and outcomes of NCDs.

## Background

Non-communicable diseases (NCDs) have become an emerging pandemic globally, with disproportionately higher rates in developing countries [1]. This trend may be attributed to a confluence of genetic, physiological, environmental and behavioural factors. The common set of lifestyle choices contributing to the rise in NCDs is the trio of unhealthy diets, physical inactivity, and tobacco use [2]. The global phenomenon of population ageing acts in tandem as older individuals have increased risk of NCDs. Without a mitigation of lifestyle behaviour, the global prevalence of NCDs is expected to continue to rise with the increase in older populations.

NCDs are the leading cause of mortality, accounting for approximately 70% of the 56 million global deaths in 2015 [3]. Four NCDs contribute to the majority of these deaths: cardiovascular diseases (17.7 million, or 45% of all NCD deaths); cancer (8.8 million, 22%); chronic respiratory disease (3.9 million, 10%); and diabetes (1.6 million, 4%) [3]. Furthermore, it is estimated that 15 million people die from a NCD between the ages of 30 and 69 years. Mortality from NCDs has a greater impact on developing countries, with 80% of premature deaths occurring in low and middle-income countries (LMICs) [4].

Cambodia, located on the Indo-China Peninsula, is one such developing country that is experiencing this demographic and health transition. An extended period of political stability provided the foundation for sustained economic growth. With approximately 7% increase in gross domestic product (GDP) per annum [5], Cambodia has emerged from a low income country to a low-middle income country. Health reforms have been on the books since the early 1990s, and have resulted in substantial gains in life expectancy. Twinned with falling fertility rates, changes to the Cambodian population structure follow those of other countries’ demographic transition to an ageing population [6]. Also mirroring trends in other developing countries, Cambodia has experienced rapid urbanisation [7–9]. The change in lifestyle and behaviours brought about by economic development, combined with rapid urbanisation and population ageing, has resulted in an increase in the incidence and prevalence of and mortality from NCDs or their risk factors. The prevalence of major NCDs in Cambodia has been increasing; diabetes prevalence was estimated to be 7% in 2010, up from 5% in 2000 [10], cancer prevalence estimated to be 0.13% in 2008 [11], while cardiovascular diseases prevalence has risen from 6% in 2000 to 6.26% in 2010 and lastly, 2.88% of the population was estimated to have chronic respiratory diseases in 2000 and has increased to 3.25% in 2010 [12]. According to the 2010 Cambodia STEPS (WHO STEPwise approach to Surveillance) survey, a nationally representative survey on the prevalence of NCD risk factors, 15% of the population is overweight, 16% have hypertension, 1 in 5 have raised total cholesterol and tobacco use is estimated at 37% [13]. While total mortality has decreased during the period, the number, as well as the proportion of deaths due to NCDs, has risen steeply. Mortality attributed to NCDs increased from 33% of the total deaths to 61% over a period of 15 years from 2000 to 2015 [14]. Specifically, cancers accounted for 13.2% of all deaths, cardiovascular disease resulted in 28.2% of all deaths and respiratory conditions caused 5.5% of all deaths in 2015 [14]. In light of the increasing exposure to risk factors, NCD morbidity and mortality are expected to rise if specific interventions to control risk factors are not implemented.

The prevention, identification, and management of NCDs are not trivial tasks. The complexity is due to the dynamic interaction between the components of NCD prevention and management and the plethora of stakeholders involved – including but not limited to policy makers, providers, patients, funders, and educators – all of whom can have different vested interests. The unique perspectives of stakeholders need to be accounted for in a common framework representing the accepted understanding of the complexities of managing and controlling NCDs.

Group model building (GMB) has been found to be a useful method in engaging different stakeholders to elicit their perspectives to address difficult and complex problems [15]. This approach combines various techniques that facilitate stakeholder engagement to develop systems models that incorporate a whole system perspective. The models are then used as an explicit representation of the cause and effect relationships that affect outcome variables, in order to gain a comprehensive insights of the complexity [16]. Stakeholder engagement through GMB allows for a coherent and comprehensive representation of important issues and insights into a single overarching framework that increases understanding of a system as a whole [17–20]. Moreover, the resultant model(s) may be used as a boundary object, or a representation of the problem that serves to facilitate further discussion among other stakeholders, bringing about a comprehensive whole system perspective [21].

This paper aims to demonstrate how systems modeling methodology of Group Model Building (GMD) can be applied for exploring and reaching consensus on non-communicable disease (NCD) management. This exercise was undertaken as a first step for developing a quantitative simulation model for generating credible estimates to make an investment case for the prevention and management of NCDs.

## Methods

GMB was developed by leaders in the System Dynamics field [15, 22–24] who recognized that the development of policy simulation models with direct active participation of stakeholders that leveraged on the iconography of system dynamics methodology introduces social dynamics that can affect the quality of model, buy-in from stakeholders, and ultimately the likelihood that recommendations from the model will be accepted and implemented. Thus, GMB refers to a system dynamics model building process in which the stakeholders are deeply and actively involved in the process of model construction through the exchange, assimilation, and integration of mental models into a holistic system description that explore questions such as: what exactly the problem we face? How did the problem situation originate? What are the underlying causes? How can the problem be tackled? [15]. The GMB approaches uses ScriptMap [25]—which is essentially standard exercises used in stakeholder engagement such as setting group expectations (hopes and fears), eliciting dynamics stories (graph over time script), eliciting causal structures (structure elicitation script), introducing system dynamics (concept model script), and finding where capacity and demand meet (ratio script)—to describe the sequences of activates in GMB research. GMB has been used significantly in health care research to address a range of issues such as; organizing healthy community program and policy initiatives [26], rising healthcare costs [27], mechanisms of primary care on health and health equity [28], understand factors that influence childhood obesity in an urban environment [29], develop new tools for chronic disease prevention and control [30]. A GMB effectiveness review assessment has been done by Rouwette and colleagues [31] which reports on a meta-analysis of findings of several studies. Also, Scott and colleagues (Scott et al. 2014) provides recent evidence on the effectiveness of GMB since earlier review by Rouwette and address three important questions: what does GMB achieve, when it should be applied and how should it be applied or improved. We applied the GMB approach to illustrate the ways in which it can be used for reaching an agreement between a large group of stakeholders with respect to NCDs prevention, screening, and treatment.

### Setting

A 2 day workshop was conducted in Phnom Penh, Cambodia, in collaboration with MOH (Ministry of Health), US Centers for Disease and Prevention (USCDC) Cambodia and World Health Organization (WHO) Cambodia. There were 25 stakeholders representing the MOH Cambodia, USCDC Cambodia, public hospitals, academic researchers, international organisations (WHO local office) and local non-governmental organisations (NGOs). These stakeholders possess both personal and institutional experience in NCD management and policy.

### Study design and participation

Group exercises were conducted with stakeholders over the 2 days to develop a preliminary qualitative model of NCDs management in Cambodia, and identify leverage points of interventions [17–20]. Exercises were planned based on a series of scripts from GMB literature [32, 33] and were led by a team of experienced facilitators. The sequence of activities were designed to build on one another (Table 1). The first session focused on participation and was aimed at discussing hopes and concerns for the 2 days. Next, stakeholders explored the outcomes of importance to a successful programme for NCDs in Cambodia and the factors responsible for promoting or inhibiting identification and treatment of individuals with NCDs in Cambodia. Finally, the participants explored the interactions and interdependences between these factors and how they influence outcomes of programmes aimed at preventing, identifying, and treating NCDs. These activities are described in more detail below.

**Table 1:** Description of group activities

Public Agenda	Team’s Activity
DAY 1	
Introduction and Overview	Background
	Purpose of the GMB conference
Hopes and Fears	Introduction of stakeholders
	Distribution of coloured sheets for writing hopes and fears
	Stakeholders are asked to share hopes and fears
	Facilitator collects the sheets and cluster on the wall
Concept Model-SD Intro	Draw bathtub with faucet and drain. Use it to explain stock and flow
	Introduce simple model of resource allocation for chronic disease prevention and treatment
	Focus on structure and behaviour relationship
Variable Elicitation	Elicit variables: distribute coloured sheets for writing problem-related variables
	Cluster the variables on board
	Vote to prioritise the variables
Reference Mode Elicitation	Stakeholders are paired to draw behaviour over time graphs of important variables
	Use separate sheets for each variable
	Stakeholders share their reference modes with the stories—facilitator probes for clarification
Structure Elicitation	Selection of initial stocks for model development
	Facilitator asks stakeholders to identify the variables that changes the stocks
	Facilitator continually probes about nature of causal relationships
	After adding couple of causal links, facilitator summarises by telling a story embedded in the model
DAY 2	
Review Model structure	Overview of model structure
Simulation demonstration	The stock and flow model developed in day 1 is translated into a calculation model
	The facilitator explains the model to stakeholders and go over few equations
	Run the model for stakeholders to observe the behaviour of some of the key outcome variables
	Engage stakeholders to suggest changes to model structure and possible future additions
Elicitation of policy options	Facilitator introduces a list of policy options from literature review
	Discuss with stakeholders to select policy options to be included in the model
Exploration of policy options	Test selected policies on the calculation model
Debriefing session	To discuss insights from the workshop and the next steps of the project

### GMB exercises

Four main interactive exercises were conducted on the first day of the workshop, and are elucidated in detail here. After introducing the agenda for the workshop to stakeholders, the participants were divided into five groups, each group was made up of individuals from different organisations. Each member of the group was given the opportunity to introduce him/herself, and state what they hope to achieve from the workshop over the 2 days, they were also asked to mention any concerns they may have. The facilitators for the workshop led the discussion on the hopes and fears for the workshop; based on the list, they clarified what was likely to be achieved over the 2 days and what might not (see appendix A). Following that, four interactive exercises were conducted:

### GMB exercise 1—Identify and prioritise outcomes

The aim of this exercise was to elicit NCD outcomes stakeholders would like to track to be able to assess if an NCD programme is successful or not. The guiding question that was presented to stakeholders to facilitate discussion was: *What outcomes of an NCD programme will tell you if the programme was successful or not?* In a round-robin fashion, after 30 min discussion among group members, each group presented one outcome at a time and the process was repeated across all groups until all outcomes identified by each group were presented. Each outcome was listed on a Post-it note and affixed to a wall. Clarifications were sought by the facilitator to ensure a common understanding of all terms. Specifically, an item was considered to be clearly defined when the participants could identify how that item would be measured. Following the presentation of the list of NCD outcomes, each stakeholder was given five stickers to vote on their top five outcomes from the combined list of outcomes from all stakeholders. Stakeholders voted by placing the stickers on the outcomes. The five outcomes with the most votes were selected as the outcomes of interest for the workshop. The list of outcomes identified by the stakeholders can be found in the Appendix B.

### GMB exercise 2—Determining causes of outcomes

The goal of this exercise was to elicit causal variables that influence the outcomes identified in the previous exercise. Stakeholders were encouraged to list all possible direct and indirect causes. Similar to the other exercises, a guiding question was used to stimulate discussion: *What causes these outcomes (as identified in GMB exercise 1) directly or indirectly?* Using an approach similar to GMB exercise 1, each group was given the opportunity to list direct and indirect causal variables. Before a variable was posted on the board, the facilitator asked clarifying questions to ensure a common understanding among the stakeholders and avoid repetition of similar ideas. An item was considered to be clearly described when the variables considered causal could be incorporated into a statement describing how the cause was hypothesised to lead to the corresponding effect. Appendix C shows the list of direct and indirect causal variables deemed by the participants to influence the outcomes of interest related to the incidence, screening and the management of NCDs identified by the stakeholders.

### GMB exercise 3—Elicit behaviour of selected NCD outcomes

In this exercise, each group was assigned one of the five outcomes from GMB exercise 1. They were asked to graph the trajectory of their outcome variable over time, from year 2005 to 2030 (a time frame agreed by the stakeholders). The graphs represented two timelines: (1) the past behaviour of the outcome variable from year 2005 to 2017 based on data, personal experience or stakeholders’ estimates; and (2) hypothesised trajectories for the likely future behaviour under two conditions: (a) business-as-usual, and (b) implementation of NCD interventions. The groups were given 30 min to deliberate and graph the behaviour over time of the outcome variables, after which each group presented their graphs to all stakeholders. Follow-up questions were asked by the stakeholders and the facilitators focusing on having each group construct a story describing the causes of the proposed trajectories. The behaviour over time of the selected outcomes elicited from the stakeholders can be found in appendix D.

### GMB exercises 4—Connect variables

This group exercise began with the presentation of a “backbone” model by the facilitators with the Cambodian population divided into five health states [34]. The five mutually exclusive health states were: (1) healthy population, (2) undiagnosed uncomplicated NCD population, (3) diagnosed uncomplicated NCD population, (4) undiagnosed complicated NCD population and (5) diagnosed complicated NCD population. The population in the healthy state was defined as individuals with no known risk factors or NCDs. Undiagnosed referred to those with one or more NCDs (either uncomplicated or complicated, but were not known to the health system and were thus not receiving requisite care). Uncomplicated NCDs corresponded to an individual who did not yet have serious disabilities, such as stroke, visual loss, kidney failure, amputation, or heart failure; those with these disabling consequences are deemed to be complicated. Individuals with uncomplicated NCDs are only likely to be known if they are screened. Those with complicated NCDs are much more likely to be known as their symptoms would often be sufficient to seek care. Population segmentation by health states has been found to facilitate service delivery and planning of care resources [34]. The transition from one health state to another (for both progressions and diagnosis) may often be different across health states [35] and is affected by various factors within the health system [36].

The stock and flow iconography of system dynamics [18] was used to show how the population flows across the five health states. The facilitators asked the group to identify the variables in the two variable elicitation exercises (outcomes and causes, listed on Post-it notes) that influenced the rate of movement of the population from one health state to another. Details of this session of the exercise is reported in the results. The concept model developed in the field with the stakeholders can be found in appendix E.

## Results

This section describes the qualitative conceptual model developed with stakeholders and the insights generated. The qualitative conceptual model was divided into two sections—prevention and treatment. The prevention section focuses on identifying NCD risk factors and the drivers for NCD screening/diagnosis; while the treatment section examines the factors that drive patients to seek NCD treatment in Cambodia.

### Prevention: Identification of risk factors and screening/diagnosis

Stakeholders identified five main risk factors that directly contribute to the development of NCDs in Cambodia. They were population ageing, genetics, tobacco use, unhealthy diets and physical inactivity. Population ageing and genetics were considered as non-modifiable risk factors. However, tobacco use, unhealthy diets, and physical inactivity were considered as modifiable factors that could benefit from public health interventions. According to the stakeholder group, physical inactivity is influenced by urbanisation (due to the relatively sedentary lifestyle in urban areas compared to rural areas) and lack of NCD knowledge (which was interpreted as lack of knowledge about the benefits of physical activity); while tobacco use decreases with urbanization. The stakeholders argued that urbanization is negatively associated with smoking because according to the 2010 Cambodia STEPS (WHO STEPwise approach to Surveillance), smoking rates in rural areas of Cambodia was higher than in urban areas. This was due to many factors: (a) rural smokers start smoking earlier than urban smokers and education may play a role in late smoking initiation among the urban dwellers; (b) average price of a pack of cigarette is 4 times higher in urban areas compared to rural areas due to more brand name cigarettes sold in urban areas; (c) better enforcement of smoking ban in urban areas compared to rural areas. Meanwhile, factors such as poverty, high income, maternal behaviour, undesired social norms and lack of NCD knowledge were identified as the main drivers for unhealthy diets. Both poverty and high income were postulated to increase unhealthy diet—which is counterintuitive. On poverty, it was argued that low income individuals do not have enough resources to maintain a healthy diet; however, high income individuals (especially those in urban areas) are increasingly consuming unhealthy fast food from the western world that is impacting on their health. In addition, poverty was found to influence unhealthy maternal behaviour (as children are fed unhealthy diet due to a lack of resources) and consequently, an unhealthy diet for the family. Lastly, undesired social norms and lack of NCD knowledge were also identified to influence unhealthy diet. According to the stakeholders, undesired social norms are influenced by advertisements that promotes unhealthy diets, while NCD knowledge was argued to be influenced by education.

**Fig. 1:**
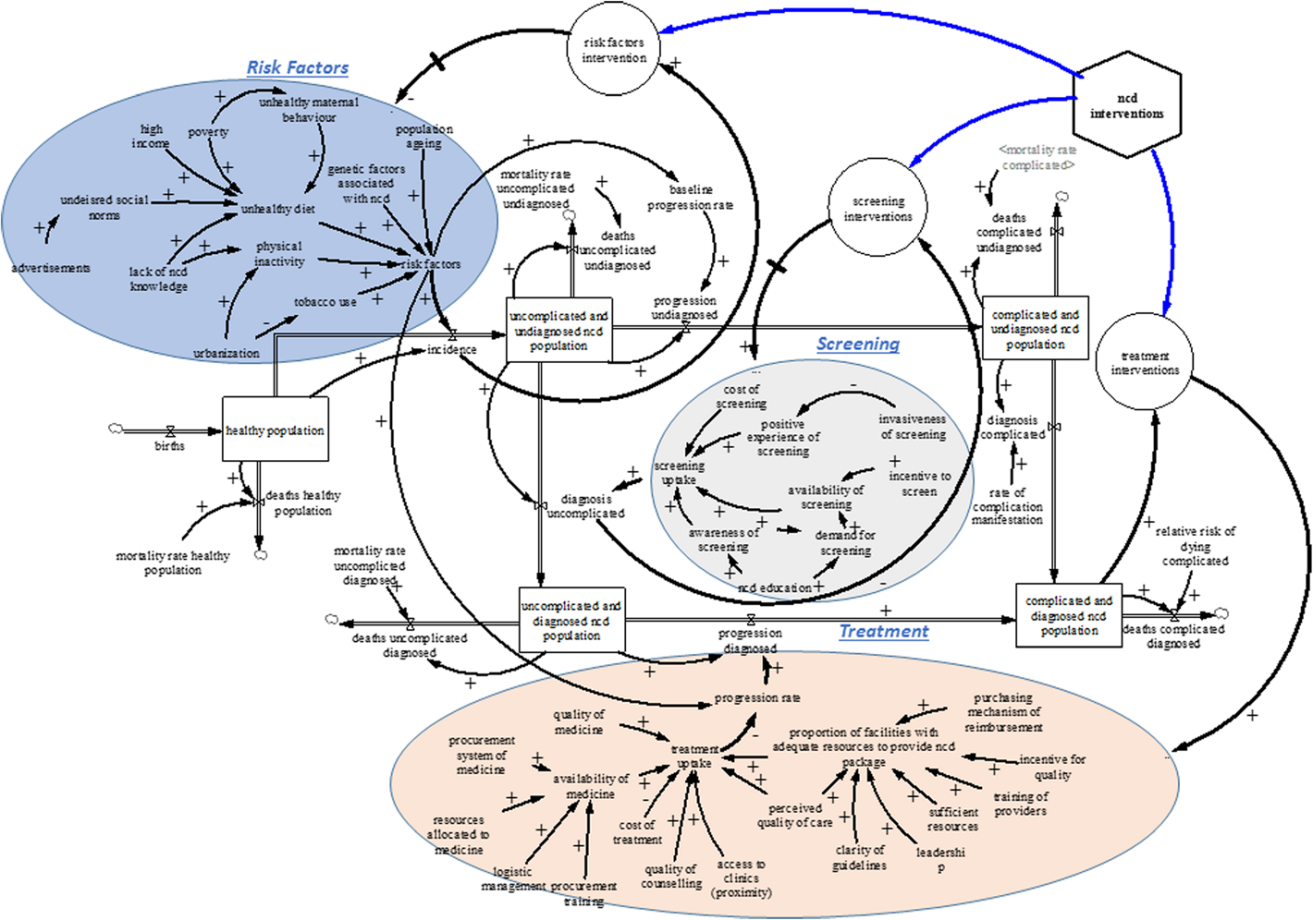
Conceptual model of NCDs prevention, identification, and management developed with stakeholders

The group then proceeded to identify factors that influence the rate at which individuals with undiagnosed NCDs get diagnosed. The main variable that was thought to change the rate of becoming diagnosed was the uptake of screening. Further discussion revealed that screening uptake is influenced by four main factors; cost of screening, availability of screening, screening experience and awareness of screening. The stakeholders thought invasiveness of screening affects the experience of those who have undergone screening, which through word of mouth may affect the likelihood of others taking up screening. Also, availability of screening services was believed to be influenced by demand for screening and the incentive for the health system in Cambodia to screen patients. Demand for screening is influenced by education about NCDs and awareness of screening. Figure 1 shows the interrelationships between the screening and diagnosis factors by stakeholders.

### NCD treatment

The last part of the exercise focused on the transition from diagnosed uncomplicated NCD to diagnosed complicated NCD. The stakeholders indicated that the average time to progress from diagnosed uncomplicated NCD to diagnosed complicated NCD depends on the proportion of diagnosed uncomplicated patients receiving adequate treatment. The proportion of the population with NCDs receiving treatment was identified as a key variable for any successful NCD program. Factors identified by stakeholders that influence the uptake of NCD treatment are (1) quality of medication, (2) availability of medicines, (3) cost of treatment (4) quality of counselling (5) access to clinics (6) perceived quality of care and (7) proportion of facilities with adequate resources to provide NCD packages. The stakeholders further expanded on the factors influencing the availability of medicines and identified the following: (1) training of staff in procurement forecasting, (2) transparent and functional logistic system, (3) resources allocated to medicines, (4) national policy for medicines and (5) procurement system for medicines. Further discussion revealed that the proportion of local healthcare facilities with adequate resources to provide ongoing treatment, in addition to the quality of treatment, is essential in keeping the population with NCDs in the diagnosed uncomplicated state for a significantly long time. This, in turn, is dependent on the following variables: (1) clarity of guidelines, (2) purchasing mechanism of reimbursement, (3) incentives for healthcare providers, (4) training of providers, (5) sufficient resources, (6) leadership and (7) service organisation. The stakeholders also expressed their belief that the proportion of facilities with adequate resources will affect the perceived quality of treatment. This is identified as a key variable that affects the percentage of diagnosed and uncomplicated population receiving treatment, which can abate progression to complicated NCDs. Figure 1 shows the interrelationships among the NCD treatment factors identified by stakeholders.

**Fig. 2:**
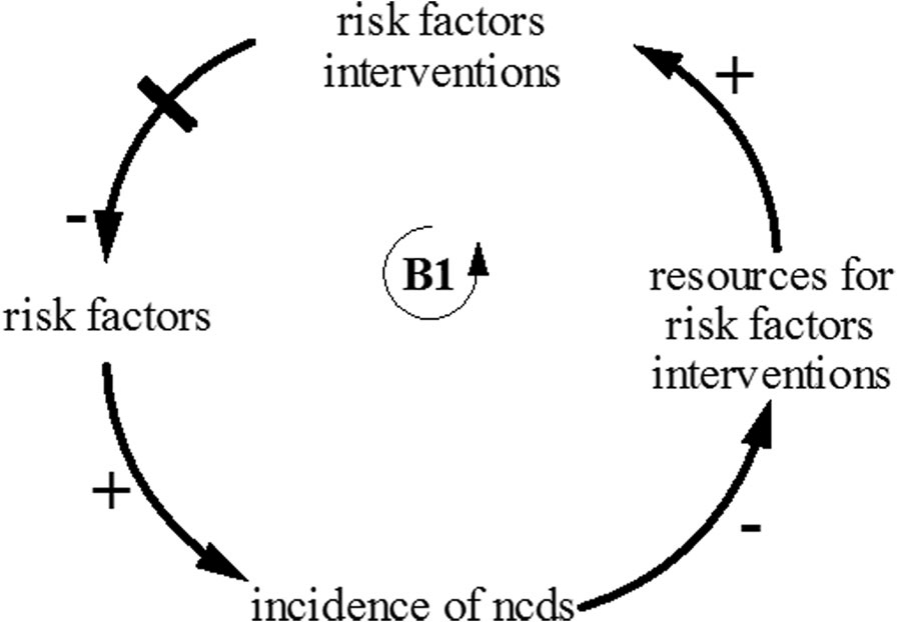
Balancing process with delay archetype—risk factors

The GMB exercise with the stakeholders generated four major insights. First, interventions on behavioural change that focuses on tackling NCDs risk factors—such as smoking, unhealthy diet, physical inactivity, and healthy aging—will not only reduce the incidence of NCDs, but also the progression from uncomplicated to complicated NCDs. However, this hypothesised cause and effect involves a significant delay. This delay between the risk factor intervention and its impact on NCD incidence and progression is likely to cause what is referred to in systems thinking as ***“balancing process with delay”*** archetype (see Fig. 2)—where resources allocated to NCDs risk factors interventions are reduced in response to the delayed observation of the impact on NCDs outcomes. If policy makers are not conscious of the significant delay, they are likely to refocus on areas that will produce immediate impact.

**Fig. 3:**
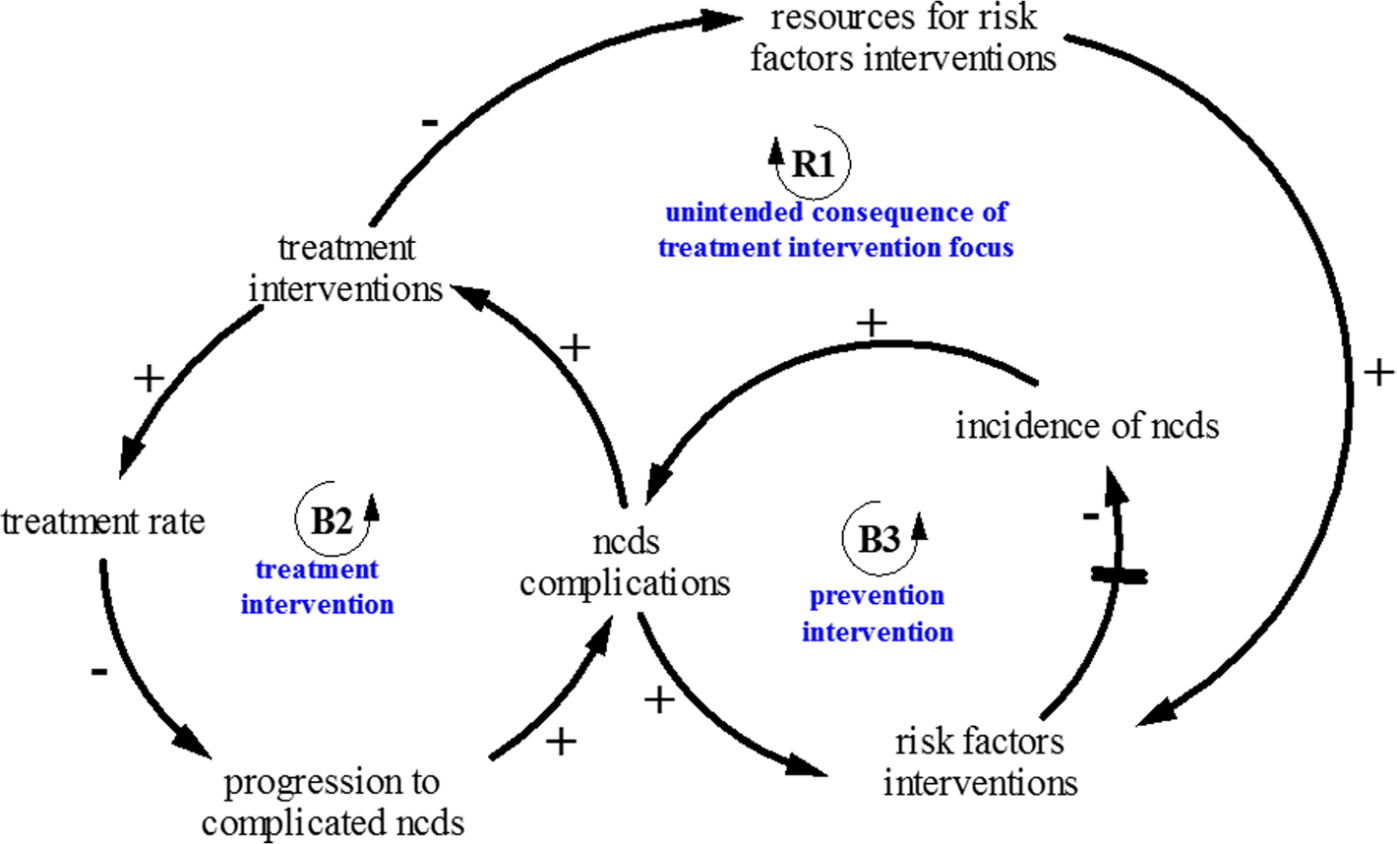
Shifting the burden archetype—screening and treatment

Second, it became apparent through the NCD model building process that screening interventions will lead to the identification of many people with NCDs who do not seek care if NCD care remains unavailable, inadequate and/or unaffordable. However, screening with adequate NCD treatment interventions which address issues of availability and affordability of NCD care will likely lead to more identification and treatment of NCDs and improve health and NCDs outcomes. As shown in Fig. 3, screening and treatment of NCDs interventions is likely to lead to the *“shifting the burden” archetype*—where screening and treatment of people with NCDs produce immediate visible consequences resulting in NCD policies that focus more and more on screening and treatment, while the more fundamental solution of prevention through risk factors reduction is used less and less. Overtime, the prevention interventions capacity declines, leading to even greater reliance on screening and treatment interventions.

**Fig. 4:**
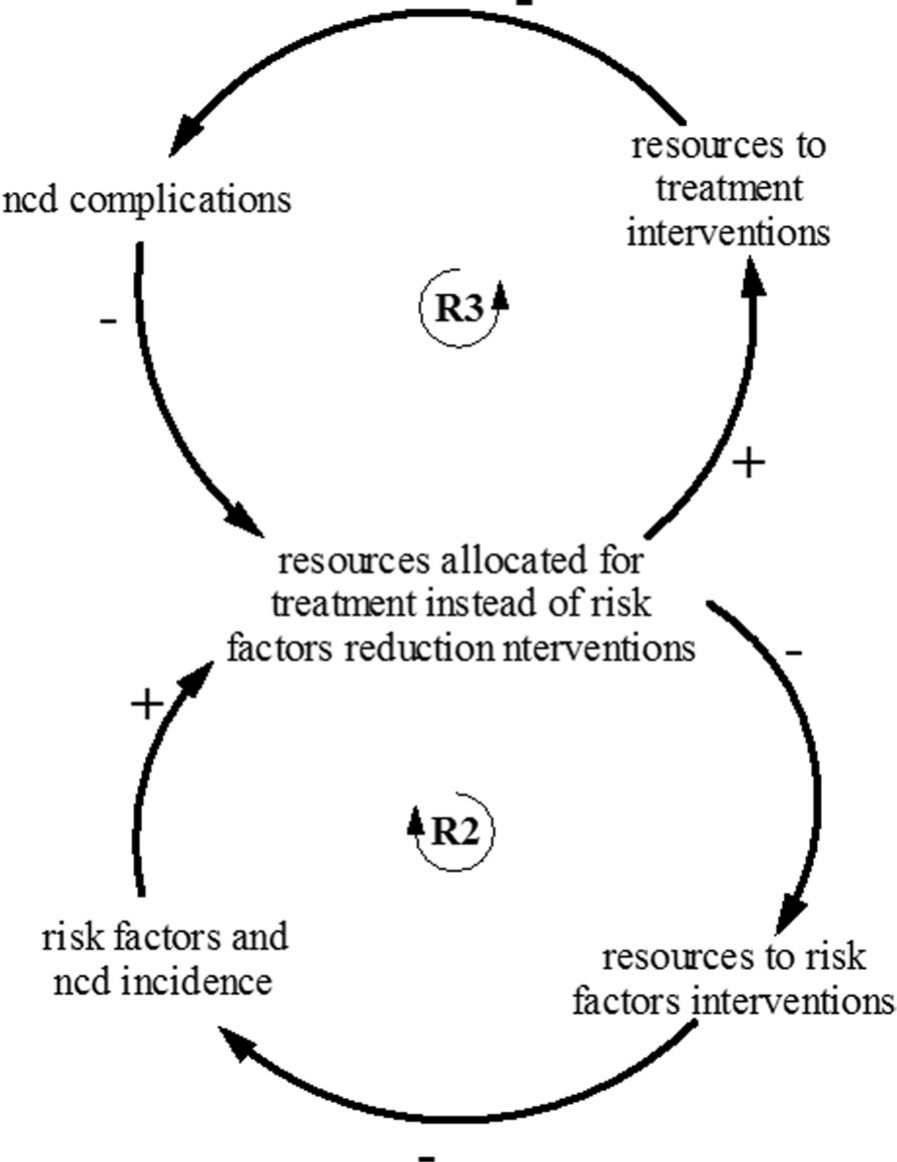
Success to the successful archetype—treatment and prevention

Third, the stakeholders realized through discussion that increasing NCD care for people with NCDs is likely to increase the prevalence of NCDs—as treatment tends to keep individuals with NCDs in stable state for longer periods thereby decreasing the probability of death from or with NCDs. One unexpected consequence is that healthcare expenditure for NCDs could increase in the future. This insight led to the identification of the *“success to the successful”* archetype (see Fig. 4)—where two interventions – prevention, and treatment – compete for limited resources. As treatment leads to an increase in the number of people with NCDs, more resources would be required to provide treatment, hence resources are diverted from prevention to treatment.

The fourth major insight achieved through the NCD model development and interactive exercises is that stakeholders had a vivid experience of how the different parts of the health system fit together as a whole to influence health and outcomes of NCDs.

## Discussion

The results from the stakeholders consultation suggesting that tobacco use, physical inactivity, unhealthy diet, genetic factors associated with NCDs and populating aging are the main risk factors of NCDs in Cambodia are supported by available evidence from developed and developing countries [2, 37]. Underlying the behaviour-related risk factors (i.e. tobacco use, physical inactivity, and unhealthy diet) are social and economic factors such as poverty, urbanization, lack of NCDs knowledge, social norms and unhealthy behaviours. These social and economic factors coupled with systemic factors such as availability, accessibility and affordability of NCD services and treatment, availability of medications, and quality of NCD care, were identified as important factors influencing care seeking behaviour of NCD patients.

According to the 2010 Cambodia STEPS (WHO STEPwise approach to Surveillance) survey, the prevalence of the behavioural factors are: tobacco use (26.4% of the population in Cambodia between the ages of 25–64 years smoke daily; of which 49.3% are men and 4.8% are women); unhealthy diet (on fruit and vegetables, 84.3% of the population between the ages of 25–64 were reportedly eating less than five servings of fruits and/or vegetables per day and therefore were considered at increased risk of developing NCDs); physical activity (10.6% of the population were classified as individuals with low physical activity, while that for moderate and high physical activity were 13.3 and 76.1% respectively). The hypothesized dynamic interactions of the factors underlying the observed behavioural risk factors of NCDs according to the stakeholders were poverty (which is more prevalent in rural Cambodia compared to urban areas and is associated with educational attainment), coupled with inadequate public health education. These were the main reasons contributing to low NCDs knowledge and awareness among the population Low knowledge and awareness of NCDs, combined with a lack of strategic policies to promote healthy behavioural change, therefore led people to engage in unhealthy behaviours such as tobacco use, physical inactivity and unhealthy diet that increase the risk of NCDs. In addition, poverty, coupled with systematic factors such as availability, accessibility and affordability of NCD treatment, availability of medications, and quality of NCD, resulted in fewer people with NCDs seeking treatment early to avoid the development of NCDs complications.

The implications of NCDs in Cambodia are manifold. An increase in NCDs will put a strain on the health system as more individuals seek care for their conditions, thereby increasing costs. Where responsive measures are not implemented, these facilities are also likely to face a shortage of human resources for health to provide adequate care. Health inequality within the country may increase as those who cannot afford to seek treatment for NCDs are likely to experience further complications in their health condition. Furthermore, increased NCD burden can result in decreased workforce productivity. In cases where NCDs are not well managed, it can lead to disability, and even premature mortality.

The insight that if policy makers are not conscious of the significant delays to observe outcomes regarding investments in NCDs (especially on prevention interventions), they are likely to refocus on areas that will produce immediate outcomes (such as treatment and screening) has important policy implications. Policy makers should be informed of the timeframe to expect outcomes of NCDs investment, to change their mental models. While some investments are likely to produce immediate outcomes, other investments are likely to impact NCDs incidence and prevalence with a long time delay. Understanding these time delays may contribute to continued support of such interventions which are fundamental to a successful NCD program. Similarly, the insight that screening and treatment of people with NCDs is likely to produce immediate visible outcomes, creating the tendency for policy makers to invest more on screening and treatment and leading to the decline of NCD prevention intervention capacity, is vital. Policy makers should consider ring-fencing some of the investment in NCDs for prevention interventions to ensure adequate and sustained resources for NCDs prevention. Lastly, the insight that as treatment for NCDs rises, the number of people under treatment, as well as the resources required to provide treatment will rise, leading to diversion of resources from prevention to treatment, further reinforces the earlier insight that policy makers should consider a fixed resource allocation for prevention to avoid crowding out prevention resources as demand for treatment rises.

Through the dynamic GMB exercises, stakeholders were actively engaged and shared their perspectives freely. This facilitated the development of a conceptual model and better understanding of the interconnections between different parts of the health system. It was generally agreed that it is imperative to start considering NCD management in the country. The understanding from this initial preliminary model about NCD incidence, prevention and management fostered an interest among stakeholders to clarify particular dynamics, and the implications of specific NCD policies that could be implemented in Cambodia.

This workshop represents a preliminary exercise, which led to some general insights on NCD management. In order to generate more useful insights that would promote an informed NCD planning process in Cambodia, credible quantitative data for inputs into a simulation model is required. The simulation model that will be generated from the qualitative model described herein needs to be further refined, incorporating views from other stakeholders, such as representatives from Ministry of Economics and Finance and patient groups. Industry partners should also be consulted and brought on board in the execution of the project. Such additional work, for example, will allow projections of the potential impact of various NCD strategies and support the case for investing in an expansion of NCD services.

## Conclusions

Managing NCDs in Cambodia will require concerted effort to tackle NCD risk factors, identify individuals with NCDs through screening and, provide adequate and affordable consistent care to improve health outcomes of people with NCDs. The GMB workshop helped to engage stakeholders with the aim to gain an in-depth understanding of NCD management in Cambodia, by drawing on their personal experiences, beliefs, and perceptions through moderated interactions. The conceptual model developed will be used to guide and inform further development of NCDs interventions nationwide.

## Appendix 1

List of hopes and fears

## Appendix 2

List of outcomes identified by the stakeholders

## Appendix 3

List of direct and indirect causal variables

## Appendix 4

Behaviour over time graphs

## Appendix 5

The concept model developed in the field with the stakeholders.

## Abbreviations

GDP: Gross domestic product
GMB: Group model building
LMICs: Low- and middle-income countries
MOH: Ministry of Health
NCD: Non-communicable diseases
NGOs: Non-governmental organisations
QALYs: Quality adjusted life years
USCDC: US Centers for Disease and Prevention
WHO: World Health Organization
